# Impact Evaluation of a Policy Intervention for HIV Prevention in Washington, DC

**DOI:** 10.1007/s10461-015-1143-6

**Published:** 2015-09-04

**Authors:** Monica S. Ruiz, Allison O’Rourke, Sean T. Allen

**Affiliations:** Milken Institute School of Public Health, George Washington University, 950 New Hampshire Ave, Suite 300, Washington, DC 20052 USA

**Keywords:** Structural interventions, Syringe exchange programs, Health policy, HIV, Injection drug users

## Abstract

Syringe exchange programs (SEPs) lower HIV risk. From 1998 to 2007, Congress prohibited Washington, DC, from using municipal revenue for SEPs. We examined the impact of policy change on IDU-associated HIV cases. We used surveillance data for new IDU-associated HIV cases between September 1996 and December 2011 to build an ARIMA model and forecasted the expected number of IDU-associated cases in the 24 months following policy change. Interrupted time series analyses (ITSA) were used to assess epidemic impact of policy change. There were 176 IDU-associated HIV cases in the 2 years post-policy change; our model predicted 296 IDU-associated HIV cases had the policy remained in place, yielding a difference of 120 averted HIV cases. ITSA identified significant immediate (B = −6.0355, *p* = .0005) and slope changes (B = −.1241, *p* = .0427) attributed to policy change. Policy change is an effective structural intervention for HIV prevention when it facilitates the implementation of services needed by vulnerable populations.

## Introduction

The District of Columbia (DC) is in the midst of a significant HIV/AIDS epidemic [1]. According to epidemiological data from the end of 2011, approximately 2.4 % of DC residents over the age of 12 years are living with HIV/AIDS [1]. Injection drug use (IDU) accounts for 14.2 % of the living cases of HIV/AIDS in the District [1]. HIV transmission through IDU disproportionately affects women and African-Americans, and the problem is most common in Washington’s most economically disadvantaged areas. Among African Americans, IDU is the third leading mode of transmission overall and the second leading mode of transmission among women [1].

The scientific community has put increased attention on the need for interventions that better address the social drivers of HIV risk [2]. Structural interventions refer to policies and programs that change environments in which health risk occurs, but without attempting to change the knowledge, attitudes, or other social interactions of persons at risk [3]. Syringe exchange is an example of a structural intervention that could have a tremendous impact on HIV prevention among people who inject drugs (PWID).

Needle and syringe exchange programs (SEPs) (henceforth referred to as “syringe exchange” or SEP) are among the simplest HIV prevention interventions for PWID, and there is copious evidence of its effectiveness both domestically and globally [4]. Expanded access to sterile injection equipment—particularly through SEP—has led to decreased needle sharing among PWID and reduced HIV incidence and prevalence [4–6], and is not associated with increased crime rates or increased illicit drug use [7]. Modeling studies have shown that widespread syringe access to active PWID for HIV prevention has societal benefit and costs less than the estimated lifetime medical costs if those persons were to become HIV-infected through shared injection equipment [8].

### Challenges in Implementing Structural Interventions

Historically, the allocation of municipal, state, or federal-level funding for any public health intervention (including SEP) is a decision governed by policy makers. Existing laws must allow for the utilization of funds in a manner that best addresses public health needs (e.g., supporting programs). Changes in legislation may be required before such structural interventions can be implemented. These policy changes—including the inclusion of seat belts in automobiles, implementation of smoke-free ordinances in buildings and public areas, and increases in alcohol taxation—have been associated with significant improvements in population level health [9–11].

Changing legislation for public health benefit is neither simple [12] nor does it happen with any consistency or frequency. In some cases, there may be a clear relationship between the source of law and its jurisdictional application (e.g., citywide smoking bans in restaurants that are limited to a specific geographic jurisdiction). In other instances, the relationships are not clear and may involve a combination of federal, state, and local laws. The DC has a particularly interesting legislative status because it is not recognized as a state and, therefore, does not have the same autonomy as other states in the Union. Instead, both Federal and local-level legislation govern DC, and it is this disconnect between Federal policy and city-level public health needs that has fueled the HIV prevention struggle for the District’s PWID.

In 1998, the United States Congress included language in the Financial Services Appropriations Bill proscribing the use of federal funds for SEP. While this legislation did not affect states and localities that wanted to use locally generated revenue, it did affect the DC because of Congress’ oversight of the city’s budget and operations through the Financial Services legislation. Thus, while the DC had a significant PWID population in need of HIV prevention services, it was the only city in the US prohibited from using municipal revenue to support syringe access. This legislative restriction became known as the “DC Ban”. Prevention Works, a community-based organization (CBO) supported through private donations and grants from non-governmental charitable foundations, operated the only SEP in the city. With the ban in place, Prevention Works was limited in its ability to secure enough funding to operate a program in a city that, at that time, had a generalized HIV prevalence of 3.0 % [13].

In December 2007, President George W. Bush signed the 2008 Financial Services Bill (HR 2764) into law. This version of the bill did not contain language prohibiting the use of locally generated revenue to support syringe access in the DC, thereby removing the DC Ban. Then-Mayor Adrian Fenty allocated $650,000 to the DC Department of Health to create the DC NEX, a program supporting several CBOs in delivering a minimum harm reduction package that includes syringe exchange, provision of condoms, referrals to HIV testing and addiction treatment, and harm reduction information [14].

The removal of the DC Ban is an instance in which a natural policy intervention occurred. The purpose of this study is to examine the impact of this policy change in Washington, DC, on IDU-associated HIV cases. Analyses were conducted to examine the actual number of new IDU-associated HIV cases observed in DC following the removal of the DC Ban and comparing it to the estimated number of infections that would have occurred had the ban remained in place. We then conducted a time series analysis to assess for changes in the numbers of pre- and post- policy change cases of IDU-associated HIV infection. We hypothesize that the lifting of the DC Ban will result in significant impact on HIV cases in DC attributable to IDU-exposure.

## Methods

The impact of the removal of the DC Ban was examined in two ways. Using autoregressive integrated moving averages (ARIMA) modeling, we first forecasted the expected number of IDU-associated HIV cases if the DC ban had remained in place and compared those to the observed number of IDU-associated HIV cases in the 24-months following the policy change. Subsequently, we used interrupted time series analysis (ITSA) to investigate significant immediate level and trend changes attributable to the policy change. ITSA is a statistical method for analyzing temporally ordered data to determine if an experimental manipulation or clinical intervention has produced a reliable change in the data [15, 16]. ITSA allows the model to account for baseline levels and trends present in the data therefore allowing us to attribute significant changes to the interruption, i.e., the lifting of the DC ban. All analyses were completed using SAS version 9.3.

Our outcome measure for both analyses was IDU-associated HIV cases; these data were obtained from the DC Department of Health’s HIV/AIDS, Hepatitis, Sexually Transmitted Diseases, and Tuberculosis Administration (DC DOH HAHSTA). HIV cases were divided into monthly observations of reported new cases of HIV attributable to either IDU or MSM/IDU exposure between August 1996 and December 2011. Due to peculiarities in HIV infection and AIDS case surveillance and reporting in the DC Department of Health during the late 1990s and early 2000s, new infections attributable to IDU exposure are represented by new AIDS cases (from 1998 onward) as well as new HIV cases (from 2001 onward).

Although the policy change that removed the DC Ban was signed into law in December 2007, the actual implementation of the policy change—i.e., when the first clean syringes were distributed through the newly created DC NEX—did not occur until May 2008. Therefore, the date of policy implementation rather than the date of policy change serves as the interruption in our ITSA model. Monthly new cases of HIV occurring prior to this implementation date constitute the pre-policy change period (n = 141) and those occurring after constitute the post-policy change period (n = 44).

Using Box and Jenkins methods [17], an ARIMA model was fitted to the pre-intervention period data. The best-fit model was identified as (0,0,1) × (0,0,1)_12_. Using this model, a forecast was created to obtain the number of IDU-associated HIV cases that would have been expected in the 24 months following the interruption date had the policy had not changed (May 2008–April 2010). The forecasted values were then compared with the actual observed cases during the same time period to calculate the number of averted cases. Next, two variables were created to assess if the implementation of the policy created a significant step-change and/or a slope change attributable to the removal of the DC Ban. The step-change was measured using a dichotomous intervention variable which assigned a 0 value to pre-policy change observations and 1 to post-policy change observations. The slope change was measured by creating a variable that assigned a 0 value to all observations in the pre-policy change time period and a 1 value to the first observation after policy implementation, but then increased the value by one in each subsequent post-implementation month (i.e., 1, 2, 3,..44). Using the model fitted to the pre-implementation period, both the step and slope change variables were entered into the model for the entire study time period as predictor variables.

Outliers with *α* < .01 were identified and corrected in the final model by adding them as input variables. Shift outliers were corrected in the model by creating dichotomous variables that assigned 0 to all observations occurring prior to the outlier date and 1 to all observations occurring after the outlier date. Additive outliers were corrected in the model by creating dichotomous variables assigning 0 to all observations other than the identified outlier that was assigned the value of 1. During model fitting, one shift and three additive outliers were added to the model. Additive outliers were identified at June 1998, July 2001, and June 2005, and a shift outlier was identified at January 1999. All four outliers were significant within the model.

This research was determined as being exempt from IRB oversight.

## Results

**Fig. 1:**
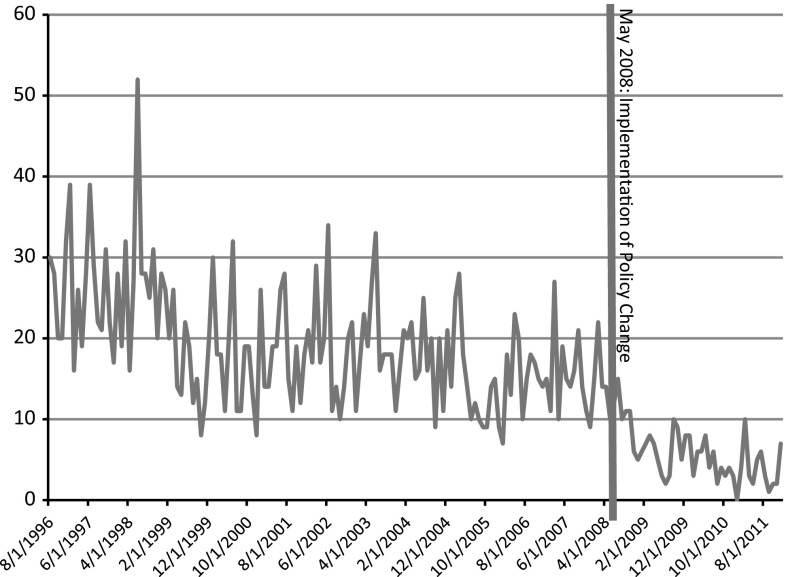
Number of HIV cases attributed to IDU or MSM/IDU exposure per month in DC between August 1996 and December 2011

Figure 1 shows the number of cases of new HIV in DC attributed to IDU by month. Visual inspection of the graph indicates a decreasing trend in new cases of IDU-associated HIV infection across the pre- and post-policy time period. The mean number of new infections attributed to IDU exposure went from 19.06 mean cases per month to 5.82 mean cases per month, a 69.5 % decrease. This same trend can be seen in both the IDU alone (16.33–4.45, 72.7 % decrease) and MSM/IDU (2.72–1.34, 50.7 % decrease) categories of IDU-attributable exposure (see Table 1).

**Table 1:** New IDU-associated HIV cases prior to and following the removal of the DC Ban

	Mean number of cases identified per month		
	Prior to policy change (8/1996–4/2008)	Following policy change (5/2008–12/2011)	Percentage change (pre- to post-policy change period
New HIV cases attributed to IDU exposure	16.33	4.45	−72.7
New HIV cases attributed to MSM/IDU exposure	2.72	1.34	−50.7
Total	19.06	5.82	−69.5

### Forecasting

**Fig. 2:**
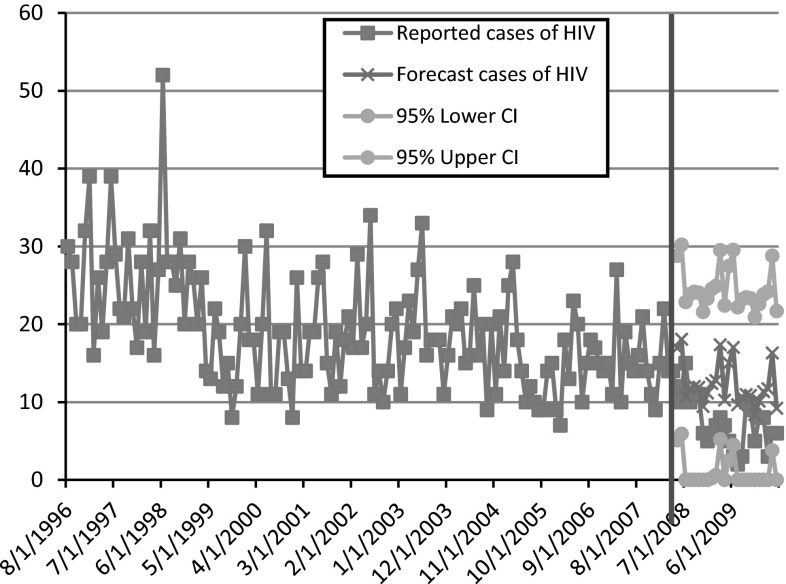
Forecasted versus observed number of new HIV cases in DC attributed to IDU exposure in the 24-month period following implementation

Using the ARIMA model fitted to the pre-implementation data, we developed a forecast of the number of expected cases for each of the 24 months following the interruption. This forecast reflects the number of cases that would have occurred each month in DC had the policy not changed. Surveillance data from the DC DOH reported 176 observed IDU-associated HIV cases in the 2 years following the repeal of the DC Ban. In contrast, the ARIMA model predicted that 296 HIV infections would have occurred had the policy remained in place. This contrast in actual versus expected HIV infections is shown in Fig. 2. Thus, the policy change allowing for municipal support of SEPs and implementation of services in the DC that occurred based on that policy change resulted in 120 averted HIV cases in 2 years.

### Epidemic Impact of Policy Change

Using the model fitted to the pre-implementation period, both the step and slope change variables were entered into the model for the entire study time period as predictor variables (see Table 2). A significant immediate and persistent step change (B = −6.0355, *p* = .0005) occurred in the month following the lifting of the ban and there was a significant reduction in slope across the post-implementation time period (B = −.1241, *p* = .0427).

**Table 2:** Interrupted time series analysis of the impact of the removal of the DC ban

	Coefficients	*t* value	*p* value
Constant	−.3312	−1.49	.1355
Baseline trend	−.1351	−1.77	.0766
Seasonal trend	.7140	10.95	<.0001
Additive outlier—June 1998	15.4411	3.39	.0007
Shift outlier—January 1999	−7.7512	−5.16	<.0001
Additive outlier—July 2001	12.6963	2.91	.0036
Additive outlier—June 2005	−11.7162	−2.66	.0078
Immediate effect of policy implementation	−6.0355	−3.48	.0005
Change in trend post-policy implementation	−.1241	−2.03	.0427

## Discussion

The findings of this research demonstrate that policy change can serve as an effective structural intervention for HIV prevention, particularly when changes in policy facilitate the creation or scale up of prevention services most needed by vulnerable and marginalized populations such as PWID. Our modeling of the forecasted versus actual epidemic curves shows that, as a result of the removal of the DC Ban, there was a 70 % decrease in the number of newly diagnosed HIV cases where reported mode of transmission was IDU. This decrease is present even after controlling for potential confounders, such as seasonality, that may have affected individuals’ risk of contracting HIV infection through injection drug use. Moreover, this decrease is within the range of findings from other studies that have examined the effects of syringe access on blood borne infections. For example, in Tacoma, syringe access was associated with a more than 80 % reduction in the incidence of hepatitis B and C infections [18]. Similarly, syringe access was associated with a 33 % reduction in HIV infection in New Haven, CT [19], and a 70 % reduction in HIV infection in New York, NY [20].

Although our forecasting only takes into account the epidemic impact of the policy change in the 2 years following the removal of the DC Ban, the evidence of epidemic impact continues to be apparent as can be seen in the ITSA which looks at the entire post-implementation period. A significant immediate and persistent drop in number of monthly cases as well as a significant decrease in trend over the 44 months following the policy implementation indicates that the lifting of the DC ban continues to have a significant impact on the number of IDU-associated HIV cases that are observed in the city. One of the limitations of ITSA as a methodology is that it does not allow for control of threats to validity within the model. In order to explain potential threats to validity, one must examine qualitative or historical data to better contextualize the results of the analyses. With regard to our ARIMA model, we observed three additive outliers that occurred in June or July of different years. These outliers are probably attributable to increases in HIV testing (and, therefore, diagnoses of new cases) that occurred around National HIV Testing Day, which is held annually on June 27th. DC HAHSTA regularly participates in National HIV Testing Day and these outliers may represent years during which the DC DOH made particularly aggressive pushes to increase testing in the District. Similarly, the shift outlier observed in January 1999 may be an artifact of the HIV awareness events that occurred in the DC around World AIDS Day 1998, which featured remarks by Secretary of State Madeleine Albright [21].

In addition to having important impact on health outcomes like HIV infection, the removal of the DC Ban has had positive impact on the cost of care for those diagnosed with HIV. According to cost-effectiveness estimates by the Centers for Disease Control and Prevention (CDC), the average lifetime cost per HIV case in 2010 was 380,000 USD [22]. Therefore, averting an estimated 120 cases of HIV infection translates to an approximate cost savings of 45.6 million USD for the DC. In the DC Appleseed Report for 2011, it was reported that the city initially funded the SEP at 650,000 USD for the fiscal year [23]. These funds were awarded to local community providers who applied through a grant program administered by the DC Department of Health and were used for all aspects of operation, i.e. staffing, clean syringes, mobile units for exchange delivery, etc. The 650,000 USD amount did not change over the next 2 years, which means that it cost the city approximately 1.3 million USD to operate the citywide SEP in the first 2 years, the same time period for which we estimated the 120 averted HIV infections. Subtracting the amount of the SEP operating costs from the total estimated lifetime cost of treating 120 cases of HIV infection decreases the overall savings to approximately 44.3 million USD. This estimated cost savings does not take into account the treatment of comorbid conditions (e.g., HCV infection, mental health conditions, etc.), but it still attests to the beneficial impact that availability and utilization of harm reduction services can have for at-risk populations such as PWID. Further, while over 91 % of DC residents overall have insurance [24], coverage is not distributed evenly and much of the burden of HIV treatment services is absorbed by publicly funded programs. To that end, it could be reasoned that the 44.3 million USD in savings is money that is saved by taxpayers.

Another way of understanding the impact of the policy change is to examine the impact that it had on the harm reduction services provided in the District. While there are no published reports on the exact number of syringes distributed in the 2 years prior to the implementation of the policy change, we obtained information from the organizational records and reports to funders of the sole SEP that was in operation during the time of the DC Ban. Based on these data, we estimate that in the two fiscal years prior to the implementation of the DC NEX in May 2008, the existing harm reduction service had distributed 180,000 clean syringes annually (personal communication, Prevention Works Board of Directors). In comparison, in FY 2009, the DC NEX reported exchanging approximately 314,000 syringes, providing 2,279 HIV tests, distributing 378,000 condoms, and linking 321 PWID to substance abuse treatment [23]. While further analyses of these data are outside the scope of this manuscript, they do show what the increased investment in syringe access “buys” in terms of actual services to the District’s PWID population.

This study had several important strengths and limitations that must be noted. With regard to the latter, the main limitation that we encountered was the quality of the available surveillance data, particularly for the earlier years of the study period on which we focused. As mentioned previously, early surveillance data reflect only AIDS cases whereas, after 2001, the data reflect both HIV and AIDS cases. Another issue is that DC HAHSTA reports that 12.3 % of diagnosed cases of HIV/AIDS (n = 1974 cases) have an unknown exposure risk [1]. For the purposes of this study we must assume that these cases are evenly distributed between each of the exposure risks. However, given how marginalized PWID populations are in society, it is possible that more than an even share of those with no exposure risk information have infections attributable to IDU. Lastly, because the DC did not adopt name-based reporting until November 2006, reported cases in the months before this may include duplicates. Data cleaning has been undertaken by the DC DOH to identify and remove as many duplicates as possible.

One of the major strengths of this study is that it is examining the public health impact of a naturally occurring policy intervention, i.e., the passage of Financial Services bill HR 2764 without the syringe exchange funding restriction (“the DC Ban”) for the DC. While this event was not naturally occurring in the classic sense of the phrase (e.g., natural disasters), it was an event that had the potential to change—and did change—the risk environment for a population vulnerable to HIV infection in the DC. In that regard, the removal of the Ban could be viewed as a policy intervention for HIV prevention for PWID. Another strength of this research is that using the ARIMA modeling allowed us to control for potential confounding factors, such as seasonality, that may have affected people’s HIV infection risk. By being able to account for the potential impact of these factors, we were able to predict with greater certainty that the reduction in IDU-associated new HIV infections was a result of the policy change and the programs that were implemented as a result of that change.

One of the critical issues surrounding the study of policy change as a structural intervention is that the potential impact of any policy change is highly dependent on how the new policies are implemented. For example, if the removal of the DC Ban were not followed by the infusion of funds by the DC City Government to create and implement a SEP network, it is likely that there would have been no tangible change in the actual availability of services for PWID and, therefore, no impact on the numbers of new infections associated with IDU. Similarly, it is important to remember that policies governing local-level operations may be overridden by policy changes at the Federal-level. This point is particularly relevant for the DC, which does not have the same autonomy as a state and therefore is more vulnerable to the types of changes that can occur when Congress authorizes legislation governing how public health interventions (such as HIV prevention efforts) can be implemented. Given the continued controversial nature of syringe exchange in many localities, it is important to understand that political processes affect the implementation of this necessary HIV prevention service. It is also important to document the evidence that supports these policy changes so that these services can be maintained and reversals in policy can be prevented.

This research provides support for the adoption of a more comprehensive and integrated approach to HIV prevention that incorporates the influence of social, structural, and policy-level factors as possible drivers of individual- and community-level risk. In showing the epidemic impact of policy change in the DC, our findings support the creation, promotion, and implementation of evidence-based policy for HIV prevention. Creating policies that are supportive of HIV prevention efforts can have substantial benefit to individuals who are vulnerable to HIV/AIDS by virtue of the environments in which they live.
